# Alterations Induced by Bangerter Filters on the Visual Field: A Frequency Doubling Technology and Standard Automated Perimetry Study

**DOI:** 10.1155/2015/909848

**Published:** 2015-01-20

**Authors:** Costantino Schiavi, Filippo Tassi, Alessandro Finzi, Mauro Cellini

**Affiliations:** Department of Specialized, Diagnostic and Experimental Medicine, Ophthalmology Service, University of Bologna, 40100 Bologna, Italy

## Abstract

*Purpose*. To investigate the effects of Bangerter filters on the visual field in healthy and in amblyopic patients. *Materials and Methods*. Fifteen normal adults and fifteen anisometropic amblyopia patients were analysed with standard automated perimetry (SAP) and frequency doubling technology (FDT) at baseline and with filters 0.8 and 0.1. *Results*. With 0.1 filter in SAP there was an increase of MD compared with controls (−10.24 ± 1.09 dB) in either the amblyopic (−11.34 ± 2.06 dB; *P* < 0.050) or sound eyes (−11.34 ± 1.66 dB; *P* < 0.030). With filters 0.8 the PSD was increased in the amblyopic eyes (2.09 ± 0.70 dB; *P* < 0.007) and in the sound eyes (1.92 ± 0.29 dB; *P* < 0.004) compared with controls. The FDT-PSD values in the control group were increased with the interposition of the filters compared to baseline (0.8; *P* < 0.0004 and 0.1; *P* < 0.0010). We did not find significant differences of the baseline PSD between amblyopic eyes (3.80 ± 2.21 dB) and the sound eyes (4.33 ± 1.31 dB) and when comparing the filters 0.8 (4.55 ± 1.50 versus 4.53 ± 1.76 dB) and 0.1 (4.66 ± 1.80 versus 5.10 ± 2.04 dB). *Conclusions*. The use of Bangerter filters leads to a reduction of the functionality of the magno- and parvocellular pathway.

## 1. Introduction

Amblyopia treatments include occlusion, optical penalization, atropine, and several pharmacological substances. All of these treatments are effective at improving visual acuity in the amblyopic eye in infancy [1–4]. Less is known about the effects of amblyopia treatments on the visual function of the nonamblyopic eye. Occlusion, which is the most common treatment for amblyopia, suppresses the entire visual input from the nonamblyopic eye [5]. Penalization produces image degradation allowing transmission of low spatial frequencies [6].

In 1960 Bangerter introduced graded translucent occlusion filters as an alternative treatment for amblyopia. Since filter density can be modulated from deep (0.1 filter) to light (0.8 filter) density and different degrees of reduction of image sharpness can be induced in the other eye, filters are considered a more gradual treatment for amblyopia compared to occlusion. The use of Bangerter filters has proven to be a useful procedure to reverse mild and moderate amblyopia [3, 4]. It is commonly believed that, contrary to occlusion, Bangerter filters can allow some binocularity to establish during amblyopia treatment, and it was recently demonstrated that the part-time use of filters can facilitate the development of motor fusion in patients with strabismic amblyopia [7]. The effects of Bangerter filters on the visual function of the sound eye have been studied with respect to visual acuity and contrast sensitivity [8]. Recently, Bangerter filters have been found to alter the visual field on standard automated perimetry (SAP) in normal eyes of adult subjects. Alterations occur in both the peripheral and central region of the visual field [9]. Nevertheless, SAP does not allow differentiation of the impairment of magno- from that of parvocellular afferent system induced by filters.

We decided to use frequency doubling technology (FDT) perimetry in visually normal adults and in adult anisometropic amblyopia patients to investigate whether the alterations induced by Bangerter filters in the visual input of the eyes to which they are applied involve the magnocellular system, especially that of a subpopulation of these retinogeniculate cells.

## 2. Materials and Methods

Thirty eyes of 15 visually normal subjects (9 female and 7 male), aged between 19 and 28 years (median 21.9 ± 2.2 years), and thirty eyes of 15 adult anisometropic amblyopia patients (10 female and 5 male), aged between 22 and 51 years (median 28.7 ± 5.9 years), were enrolled in the study.

All participants underwent a complete ophthalmological evaluation, including best-corrected visual acuity (BCVA) measurement, Goldmann applanation tonometry, slit lamp examination of the anterior and posterior segment, cover test, and random-dot stereopsis evaluation. All normal subjects had normal binocular vision with random-dot stereopsis, absence of retinal and optic nerve pathologies, and transparent dioptric media. None of them had a history of strabismus or amblyopia.

The amblyopic patients had unilateral anisometropic amblyopia and absence of binocular vision with random-dot stereopsis, absence of retinal and optic nerve pathologies, and transparent dioptric media. We excluded patients affected by strabismus and with a visual acuity of the amblyopic eye less than 0.6 decimals. Inclusion criteria for the anisometropic amblyopes were as follows: patients had a difference in refractive error between the eyes of 1.5 D S.E. or more and a visual acuity higher than 0.6 in the amblyopic eye.

BCVA was measured at a distance of 5 metres with “E” charts, first without filters and then with 0.8 and 0.1 filters.

The visual field was tested in both eyes of all subjects with both SAP and FDT perimetry at baseline, and then sequentially with 0.8 and 0.1 Bangerter filters (Bangerter filters, Ryser Optik, St. Gallen, Switzerland).

The same pair of 0.8 and 0.1 filters was used for all subjects in order to prevent any variation in density between filters of the same power.

SAP tests were performed using the Humphrey Field Analyser II (Carl Zeiss, Meditec, Dublin, CA, USA) and the SITA standard 30.2 program.

The FDT Visual Field Instrument (Welch Allyn, Skaneateles Falls, NY, USA) was used for FDT perimetry tests. Global indices, that is, the mean defect (MD) and the pattern standard deviation (PSD) resulting from SAP and FDT perimetry, were used for comparison between tests and for statistical analysis.

FDT perimetry is a technique designed for the rapid and effective identification of visual field impairment in glaucoma patients [10]. The FDT stimulus consists of a bar grid with a low-frequency spatial sinusoidal profile (0.25 cycles/degree) subjected to a sinusoidal temporal commutation at a frequency of 25 Hz. FDT perimetry is based on the principle of the frequency-doubling illusion, in which the subject perceives twice the number of bars that are actually present [11]. The cells that present a nonlinear response to the contrast in the test image, which are therefore responsible for this illusion, are a subgroup of M cells [12]. FDT perimetry tests were performed using the N-30 full-threshold program. In these tests, target stimuli consisted of individual sinusoidal gratings, 10 degrees square at 0.25 cycles/degree, alternately flashing at 25 Hz. Targets were in one of the 19 areas within the central 30 degrees of the visual field. For each visual field, we evaluated the mean defect (MD) and the pattern standard deviation (PSD).

SAP tests were performed with the full optical correction in all subjects, while FDT perimetry tests were carried out without optical correction since all eyes presented refractive errors that did not exceed a Spherical Equivalent (S.E.) of 4 dioptres (D).

None of the study subjects had previous experience with SAP or FDT perimetry.

Participants in the study underwent in three different sessions at intervals of 7 ± 2 days visual field tests with either SAP or FDT perimetry in both eyes in basal conditions, that is, without filters. Later, we tested the participants with both SAP and FDT perimetry in three different sessions at intervals of 7 ± 2 days first without filters (1st session), then with filter 0.8 (2nd session) and filter 0.1 (3rd session). The right eye was the first examined eye in the healthy subjects, whereas the sound eye was tested before the amblyopic eye in the amblyopes. The results of this latter test series were considered for the statistical analysis.

Data were analysed using the MedCalc 10.9.1 statistical program (MedCalc Software, Ostend, Belgium). MD and PSD values obtained by testing the visual field with SAP and FDT at the baseline and after applying Bangerter's filters 0.8 and 0.1 were statistically analysed within groups (control group, amblyopic eye, and sound eye) and among the groups separately using Wilcoxon's test and ANOVA test for the analysis of variance, considering as significant *P* < 0.05.

The tenets of the Declaration of Helsinki were followed, and, after a full explanation of the aim of the study and of the procedures, all participants signed a written informed consent.

## 3. Results

The mean BCVA in decimal notation without filters was 1.0 ± 0.2 in the control group and 1.0 ± 0.1 in sound eyes, respectively. The mean refractive error was 0.75 ± 1.5 D (range: −4 to +2.5 D S.E.) in both groups.

The mean BCVA in amblyopic eyes was 0.7 ± 0.2. Best corrected visual acuity in basal condition and with application of 0.8 and 0.1 Bangerter's filters did not differ significantly between controls and sound eyes, whereas there was a significant difference when matching the control group and sound eyes against amblyopic eyes (Table 1).

The results were analysed taking separated the two perimetric methods used.

### 3.1. Standard Automated Perimetry (SAP)

The intergroups statistical analysis shows that MD at baseline was statistically significantly decreased in amblyopic eyes (*P* < 0.020) compared with controls. Sound eyes presented a decrease in MD, but this was not statistically significant either compared to controls (*P* < 0.089) or in comparison to the amblyopic eyes (*P* < 0.224).

After the application of 0.8 filter, we observed a statistically significant decrease (*P* < 0.027) of MD values in the amblyopic eyes compared to controls.

Furthermore, the sound eyes presented a decrease in MD value that was not statistically significantly different when compared with controls (*P* < 0.145), and there was not a statistically significant difference in MD decrease when comparing the amblyopic eyes with the sound eyes (*P* < 0.109).

With 0.1 filter there was a statistically significant decrease of MD compared with controls in either the amblyopic (*P* < 0.050) or sound eyes (*P* < 0.030). On the other hand, there was not a statistically significantly different decrease of MD when comparing amblyopic and sound eyes (*P* < 0.926) (Table 2).

In all tested conditions, that is, with filters 0.8 and 0.1, the PSD was statistically significantly increased in the amblyopic eyes and in the sound eyes compared with healthy eyes, while there were not statistically significantly differences between amblyopic eyes and sound eyes (Table 2).

The intragroup statistical analysis shows that in every group there was a significant alteration of both PSD and MD after the interposition of filters not only compared to baseline, but also between filters 0.8 and 0.1 (Tables 3 and 4).

### 3.2. Frequency Doubling Technology (FDT)

The intergroups statistical analysis shows that MD was decreased, in all tested conditions, in the amblyopic eyes and in the sound eyes compared to healthy eyes, while there were no differences between amblyopic eyes and sound eyes (Table 2).

The PSD at baseline was statistically significantly increased in both the amblyopic (*P* < 0.013) and sound eyes (*P* < 0.001) compared to the healthy controls. On the other hand, there were no statistically significant changes in all other tested conditions between groups.

The intragroup statistical analysis shows that in every group there was a statistically significant decrease of MD after the interposition of filters not only compared to baseline, but also between filters 0.8 and 0.1 (Table 5).

The PSD values in the control group were statistically significantly increased with the interposition of the filters compared to baseline but no statistically significant difference was found between filters 0.8 and 0.1, whereas we did not find statistically significant changes in the amblyopic and sound eyes compared to baseline or by comparing 0.8 filter with 0.1 filter (Table 6).

## 4. Discussion

Bangerter filters are graded translucent filters used to treat amblyopia in children [2, 3]. The filters vary in density and are intended to induce progressive degradation of distance optotype visual acuity and other visual functions, including near optotype acuity, Vernier acuity, stereopsis, and contrast sensitivity [6, 8, 13]. Magnified inspection of Bangerter filters reveals that they consist of a characteristic pattern of microbubbles, the number of which in each filter is related to the degree of visual degradation [6]. The filter label indicates the decimal acuity predicted by the manufacturer when the filter is placed in front of eyes with normal visual acuity. In our study we found that the 0.8 filter reduced visual acuity to 0.5 ± 0.2, while the 0.1 filter reduced visual acuity to 0.3 ± 0.1 in healthy subjects. These data seem to confirm the variability in visual acuity obtained from the use of filters reported by previous studies [14, 15].

The use of filters 0.8 and 0.1 significantly reduced visual acuity in both the amblyopic and sound eye, but this reduction was not statistically significant in the intergroups analysis.

To avoid the influence of a learning effect and a fatigue effect, and in order to minimize intrasession test-retest variability which is well known to occur in SAP and FDT, participants in the study underwent in three different sessions at intervals of 7 ± 2 days visual field tests. The right eye was the first examined eye in the healthy subjects, whereas the sound eye was tested before the amblyopic eye in the amblyopes to eliminate the fatigue effect that may worsen the perimetric indexes of the sound eye [16–18].

The decrease of MD index is related to the reduction of visual acuity as was found in other diseases that reduce the transparency of the anterior segment, for example, cataract [19].

The Bangerter filters produce monotonically increasing attenuation of the higher spatial frequencies [6] and they leads to a perimetric alteration with the occurrence of a central scotoma that is greater (o deeper) for more penalizing filters [9].

**Figure 1:**
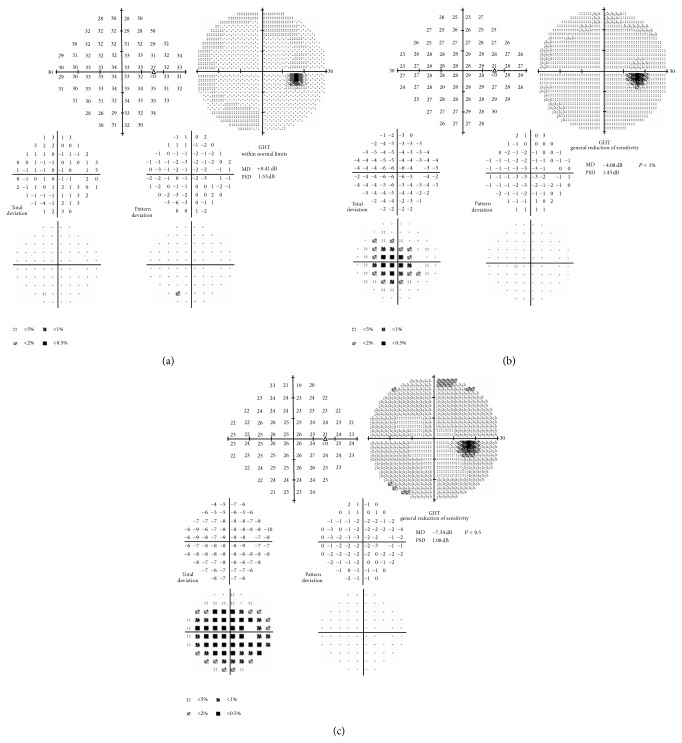
The visual field evaluated with SAP in normal conditions (a), with 0.8 (b) and 0.1 Bangerter filters (c).

**Figure 2:**
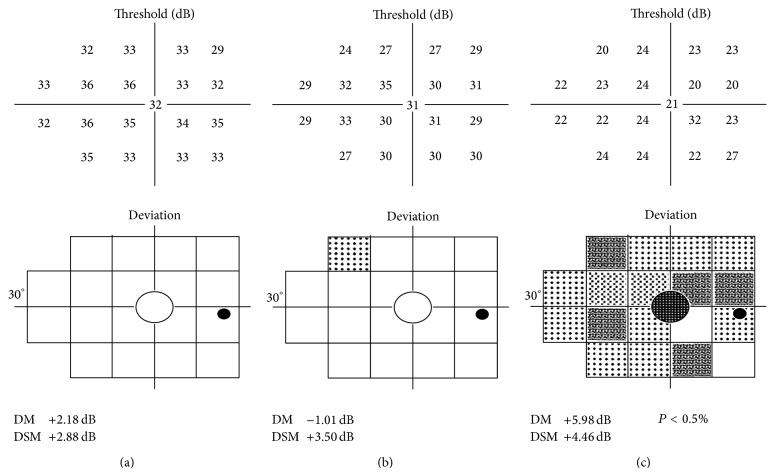
The visual field evaluated with FDT perimetry in normal conditions (a), with 0.8 (b) and 0.1 Bangerter filters (c). DM: MD (mean deviation); DSM: PSD (pattern standard deviation).

In our study we found that the use of filters with SAP caused a scotoma that was limited to the central portion of the visual field with the 0.8 filter, while it extended to the entire central 30° with the 0.1 filter (Figures 1 and 2).

This effect of Bangerter filters should be a consequence of a specific action of filters that in particular penalizes the parvocellular retinogeniculate system (i.e., the P-cells), which is responsible for central visual acuity and the discrimination of visual details [20].

Porciatti and Ventura [21] used PERG to show that Bangerter filters, besides reducing contrast stimuli [6], also suppress the square-wave edges of the grating pattern, thereby removing the high-spatial frequency content of the stimulus. Since P-cells have smaller receptive fields compared to M-cells, blurring or reducing the stimulus's spatial frequency is expected to shift the relative contribution of M and P generators towards M generators with shorter latency [22, 23].

Retinal ganglion cells (RGC) are commonly divided into two major classes: M-cells (parasol cells), with large dendritic field sizes and projecting to the magnocellular layers of the lateral geniculate nucleus, and P-cells (midget cells) with small dendritic field sizes and projecting to the parvocellular layers of the lateral geniculate nucleus. P-cells respond preferentially to intermediate and high spatial frequencies and to slow or stationary targets, whereas M-cells respond better to low spatial frequency stimuli, high achromatic flicker, and fast movement [24, 25]. M-cells are much more sensitive to luminance contrast than P-cells [26], and their response has faster temporal dynamics than P-cells [27]. Electrophysiological studies demonstrated that low contrast stimuli elicit a relatively larger contribution of M generators compared to high contrast stimuli [28].

FDT perimetry [29, 30] is based on the frequency-doubling illusion [10, 31] that occurs when the subject views a counter-phased grating with a low spatial frequency and a high temporal rate. The perception is double the spatial frequency of the actual physical grating [31]. This illusion has been attributed to a subset of the magnocellular ganglion cells, which are nonlinear in their response properties [32].

Maddess and colleagues [22, 33] attributed this phenomenon specifically to the M-y ganglion cells, a subset of approximately 3-4% of the magnocellular mechanisms that exhibit nonlinear response properties and have large-diameter fibres [34].

In previous studies the FDT-PSD has proved to be very sensitive in highlight early damage of the M-y ganglion cells in diseases that damage the visual pathway, for example, ocular hypertension and early glaucoma [35].

With FDT perimetry we found that the application of filters in the healthy eye causes a significant increase in the PSD values. On the contrary, filters did not cause statistically significant changes of PSD values in the amblyopic eye and in the sound eye.

In the amblyopic and sound eyes Bangerter filters seem not to alter the retinal sensitivity, which is already altered in basic condition compared to baseline values, as shown by the analysis of variance (Table 5).

On the other hand, in normal subjects filters lead the healthy eyes to a condition like amblyopia.

Changes of FDT-PSD confirm that in the amblyopic eye there is an alteration not only of parvo- but also of magnocellular system, as demonstrated by the deficits in velocity discrimination, saccadic eye dysfunction, and alteration of contrast sensitivity [36–39].

It would seem that amblyopic patients present a condition of generalized reduction of functionality not only of the parvocellular cells, but also of a subpopulation of M-cells (the M-y cells) that are sensitive to the variation of contrast. This dysfunction of the retinogeniculate pathways in monocular amblyopia involves also the so-called “sound eye” that, if normal, should not behave after application of filters as an amblyopic one [40].

This seems to confirm the results of electrophysiological experimental studies [21].

The results of this study open new perspectives in the knowledge of the physiology and pathology of the afferent visual system.

## Figures and Tables

**Table 1:** BCVA values (mean values and standard deviation) without filters (base) and with 0.8 and 0.1 Bangerter filters.

		Control group	Amblyopic eyes	*P* < 0.05	Sound eyes	*P* < 0.05^*^	*P* < 0.05^**^
BVCA (decimal)	Baseline	1.0 ± 0.2	0.7 ± 0.2	0.0001	1.0 ± 0.1	0.328	0.0001
	0.8	0.5 ± 0.2	0.4 ± 0.1	0.002	0.5 ± 0.1	0.424	0.0001
	0.1	0.3 ± 0.1	0.2 ± 0.1	0.004	0.3 ± 0.1	0.376	0.0001

^*^Control group versus sound eyes.

^**^Amblyopic eyes versus sound eyes.

**Table 2:** Perimetry values (mean values and standard deviation) without filters (baseline) and with 0.8 and 0.1 Bangerter filters.

		Control group	Amblyopic eyes	*P* < 0.05	Sound eyes	*P* <0.05^*^	*P* < 0.05^**^
SAP-MD	Baseline	−1.60 ± 1.00	−3.01 ± 1.54	0.020	−2.79 ± 2.06	0.089	0.224
	0.8	−4.56 ± 0.76	−5.73 ± 3.24	0.027	−4.91 ± 3.26	0.145	0.109
	0.1	−10.24 ± 1.09	−11.34 ± 2.06	0.050	−11.34 ± 1.66	0.030	0.926

SAP-PSD	Baseline	1.35 ± 0.24	1.74 ± 0.48	0.004	1.60 ± 0.26	0.006	0.244
	0.8	1.51 ± 0.19	2.09 ± 0.70	0.007	1.92 ± 0.29	0.004	0.359
	0.1	1.85 ± 0.72	2.68 ± 0.78	0.004	2.47 ± 0.99	0.035	0.263

FDT-MD	Baseline	−0.96 ± 0.71	−3.42 ± 3.38	0.005	−2.65 ± 3.11	0.050	0.126
	0.8	−2.35 ± 2.37	−5.12 ± 4.37	0.023	−5.34 ± 4.01	0.014	0.430
	0.1	−6.37 ± 2.41	−10.47 ± 3.17	0.002	−10.14 ± 3.77	0.006	0.870

FDT-PSD	Baseline	3.15 ± 0.62	3.80 ± 2.21	0.013	4.33 ± 1.31	0.001	0.353
	0.8	4.06 ± 1.15	4.55 ± 1.50	0.329	4.53 ± 1.76	0.353	0.363
	0.1	4.71 ± 0.77	4.66 ± 1.80	0.926	5.10 ± 2.04	0.743	0.611

^*^Control group versus sound eyes.

^**^Amblyopic eyes versus sound eyes.

MD: mean deviation; PSD: pattern standard deviation; SAP: standard automated white-on-white perimetry; FDT: frequency doubling technology perimetry.

**Table 3:** Statistical analysis using ANOVA for repeated measurements, SAP-MD on altering caused by the application of Bangerter filters 0.8 and 0.1 in control group and amblyopic and sound eyes.

	MD Control baseline	MD Control 0.8	MD Control 0.1	MD Amblyopic 0.8	MD Amblyopic 0.1	MD Sound 0.8	MD Sound 0.1
MD control baseline	∗∗∗	0.0001	0.0001				
MD control 0.8		∗∗∗	0.0001				
MD amblyopic baseline			∗∗∗	0.0017	0.0001		
MD amblyopic 0.8				∗∗∗	0.0001		
MD sound baseline					∗∗∗	0.0141	0.0001
MD sound 0.8						∗∗∗	0.0001
MD sound 0.1							∗∗∗

**Table 4:** Statistical analysis using ANOVA for repeated measurements, SAP-PSD on altering caused by the application of Bangerter filters 0.8 and 0.1 in control group and amblyopic and sound eyes.

	PSD Control baseline	PSD Control 0.8	PSD Control 0.1	PSD Amblyopic 0.8	PSD Amblyopic 0.1	PSD Sound 0.8	PSD Sound 0.1
PSD control baseline	∗∗∗	0.019	0.035				
PSD control 0.8		∗∗∗	0.205				
PSD amblyopic baseline			∗∗∗	0.009	0.001		
PSD amblyopic 0.8				∗∗∗	0.021		
PSD sound baseline					∗∗∗	0.002	0.003
PSD sound 0.8						∗∗∗	0.013
PSD sound 0.1							∗∗∗

**Table 5:** Statistical analysis using ANOVA for repeated measurements, FDT-MD on altering caused by the application of Bangerter filters 0.8 and 0.1 in control group and amblyopic and sound eyes.

	MD Control baseline	MD Control 0.8	MD Control 0.1	MD Amblyopic 0.8	MD Amblyopic 0.1	MD Sound 0.8	MD Sound 0.1
MD control baseline	∗∗∗	0.011	0.0001				
MD control 0.8		∗∗∗	0.0001				
MD amblyopic baseline			∗∗∗	0.042	0.0001		
MD amblyopic 0.8				∗∗∗	0.0001		
MD sound baseline					∗∗∗	0.0003	0.0001
MD sound 0.8						∗∗∗	0.0001
MD sound 0.1							∗∗∗

**Table 6:** Statistical analysis using ANOVA for repeated measurements, FDT-PSD on altering caused by the application of Bangerter filters 0.8 and 0.1 in control group and amblyopic and sound eyes.

	PSD Control baseline	PSD Control 0.8	PSD Control 0.1	PSD Amblyopic 0.8	PSD Amblyopic 0.1	PSD Sound 0.8	PSD Sound 0.1
PSD control baseline	∗∗∗	0.0004	0.0010				
PSD control 0.8		∗∗∗	0.0982				
PSD amblyopic baseline			∗∗∗	0.177	0.239		
PSD amblyopic 0.8				∗∗∗	0.857		
PSD sound baseline					∗∗∗	0.195	0.674
PSD sound 0.8						∗∗∗	0.112
PSD sound 0.1							∗∗∗
